# Exploring the Organizational Culture in Adult Day Services (ADS) and Its Effect on Healthcare Delivery in Taiwan

**DOI:** 10.1155/2020/4934983

**Published:** 2020-02-13

**Authors:** Chih-Ling Liou, Mary Dellmann-Jenkins

**Affiliations:** ^1^Department of Human Development and Family Studies, Kent State University at Stark, North Canton, OH 44721, USA; ^2^Department of Human Development and Family Studies, Kent State University, Kent, OH 44242, USA

## Abstract

Studies conducted in nursing homes/hospitals have shown that organizational culture plays an important role in care delivery and group culture leads to better quality of care. To explore the organizational culture and care delivery in adult day services (ADS) centers in Taiwan, we used both quantitative and qualitative research methods. Quantitative data from the Competing Values Framework (CVF) assessment showed that the group culture was dominant at all three centers. Qualitative data from observation and staff interviews uncovered both group and nongroup cultural elements. The group cultural elements, such as flexible management, teamwork environment, and sharing the same values, contributed to good care; however, the nongroup cultural elements, such as the staff-centered view, hierarchy, and conflicts within the leadership, led to negative staff-staff and staff-clients interactions. Further research is needed to untangle the complexity between quality care delivery and organizational culture.

## 1. Introduction

Taiwan, like other developed Asian countries, has experienced rapid growth in its senior adult population. The government estimates that the percentage of individuals aged 65 and over in Taiwan is more than twice that of European countries and United States [1]. With a higher proportion of the population reaching advanced ages, the number of older people needing assistance with personal care and daily activities will increase. According to the 2013 National Health Interview Survey, around 86% of older adults in Taiwan have at least one chronic condition [1]. The Ten-Year Long-Term Care Project 2.0 [2] reported that the number of senior adults in Taiwan receiving long-term care (LTC) services was 416,473 people (75% of the total LTC recipients) in 2017 and will grow to 623,583 (80% of the total LTC recipients) in 2026.

Influenced by Chinese culture, care for frail older people in Taiwan has been provided mostly by family [3]. Although cultural norms about caring for aging relatives are changing [4], according to Chiu's [5] survey results, 73% of the older adults in Taiwan still consider being cared by and living with adult children as the ideal arrangement. Currently, 63% of Taiwanese older adults with disabilities live at home and are taken care of by family members [2]. However, Taiwan has experienced a rapid family-structure change from 3.57 people per family in 1996 to 2.77 people per family in 2015 [2]. The nuclear family, composed of two adults and dependent children, has become the dominant family structure in Taiwanese society. Families are being challenged with meeting and balancing the demands of childcare and providing care to elderly relatives simultaneously [3]. Family caregivers in Taiwan are spending substantially more time (about 13.5 hours per day) providing home services to elderly members compared with the time they would normally spend in the workplace (i.e., 8-hour workday) [3]. Many adults in Taiwan report that their caregiving roles have led to feelings of depression, inability to balance their careers with elder care responsibilities, and/or financial problems due to the impact of caregiving [3].

In order to meet elders' preferences to live with family members and reduce family burden associated with home care, the Taiwanese government passed a 10-year long-term care plan (2007–2016) to promote an “aging-in-place” community care policy by integrating community and home care resources to achieve the goal of caring for older adults in their homes/communities [3]. Community-based adult day care services (ADS), providing care for people with Alzheimer's disease and other dementia or/and physical disability, have grown drastically from 31 centers in 2008 to 178 centers in 2016 [2]. The government planned to spend 10 billion Taiwanese dollars (equal $343,400,000) to support each town having at least one ADS center [6].

While the Taiwanese government aims to provide ADS centers to meet the needs of the increasing numbers of people with dementia and/or physical disabilities [2], there is limited research on the organizational culture and care delivery in adult day services (ADS) centers in Taiwan. Past studies have focused primarily on ADS usage and/or effectiveness for family caregivers and the older adults [7] or on specific activities in ADS [8, 9]; few have focused on care performance or quality of care at ADS centers in Taiwan.

Quality of care is considered an important factor in LTC services effectiveness; however, the definitions and measures for quality of care vary [10]. Although the clinical aspect of quality of care (e.g., hospitalization, nutrition status, and functional status) is still the primary consideration, there is a shift toward the social aspect of care and taking into consideration human relation dimensions and interpersonal relationships [11, 12]. Researchers have demonstrated that interpersonal relationships with staff influence the experience and quality of life of residents in nursing homes [13]. Studies have also recognized organizational culture as a relevant influence on forming relationships and affecting the quality of care across health and social care settings [14–17].

Studies conducted internationally have shown that organizational culture plays an important role in affecting care delivery, quality of care, and residents'/patients' experiences in the healthcare setting [12, 16–21]. Van Beek and Gerritsen [17] collected data in 11 nursing homes in the Netherlands and found that in a facility characterized by a group culture, better quality of care was provided and staff interacted with residents in a dynamic fashion, adapting to residents' responses and individual concerns. Nakrem [12] conducted research at four nursing homes in Norway and concluded that residents living in nursing homes with distinct cultures/orientations experienced their lives at facilities differently. Killett et al. [16] observed 11 care homes in UK and identified organizational culture as an important influence on the quality of care provided. Finally, with respect to older adults living with dementia, the importance of organizational culture has long been recognized as a key contributor in the provision of good care [16]. Kitwood [22] emphasized that the interactions between staff and people with dementia did not derive from the staff's intention but rather from the care culture at the facility.

Most of the research studies on organizational culture have been carried out in nursing homes/hospitals in western countries. The role of organizational culture at ADS centers in non-Western society is relatively unknown. MacLachlan [23] indicated that different cultures contribute to the complexity of care delivery and talked of the need for a global health perspective. The goal of the present study is to explore the organizational care culture within three ADS centers in Taiwan, which led to our research questions, “What is the organizational culture at Taiwanese ADS centers and how does the organizational culture affect care delivery at center?” The findings of our study begin to fill a gap in the literature on understanding the role organizational culture plays in affecting the caregiving priorities and practices of administration and staff in adult day care centers in Taiwan.

## 2. Methods

The present study used the Competing Values Framework (CVF) assessment, which has a well-established reliability as demonstrated by previous research [14, 17], is often used in healthcare research [18, 24, 25], and is one of the few comprehensive culture instruments used to compare culture across organizations [24]. The CVF assesses the dominant organizational culture based on four culture types: group, developmental, hierarchy, and market culture [14].

In addition to the CVF assessment, we adopted focused ethnography, which seeks to understand and interpret the behaviors of groups of people [26], as our research method to examine the relationship between organizational culture and care delivery. Focused ethnography with the combination of interview and observation methods would allow integration of expressed values, behaviors, and practices [21]. ADS clients with dementia are often not consulted about their care receiving experiences but are likely to be highly sensitive to the artifacts of organizational culture [16]. Focused ethnography, collecting data with interviews and participant observation in a short but intensive time period, would optimally offer critical insights into ADS care for people with dementia [16]. Data were gathered after the Institutional Review Board (IRB) of the sponsoring university approved the human subjects' protections and data procedures. This study is part of a larger research project exploring the organizational culture and quality of care at ADS centers by collecting data on the perspectives of staff members and clients. In the present study, only the staff members' responses to the CVF assessment and their interviews were used along with observations on staff-client and staff-staff interactions.

### 2.1. Settings

All three ADS centers were located in the same city of Taiwan because it has a high percentage of the senior adult population (14.7%) [2]. The first author was also familiar with this city because it was her hometown. The first author purposively selected these three centers based on their heterogeneity in organizational characteristics. All three centers belonged to a social model of ADS (which is the major type of ADS in Taiwan normally located in the community and supervised by the Department of Social Affairs) and were funded by the government. Each center was run by a different private, not-for-profit organization and possessed distinct care philosophies and caregiving/service goals, which may lead to different care cultures. Each center is described below with no specified names to protect the confidentiality of participants. See Table 1 for more information on the three centers.

*Center A* is owned by a large, local nursing home organization and promoted for its therapeutic activities, unit care, and the physical design. There were 48 clients enrolled with one director, two registered nurses (RNs), one full-time social worker and one part-time social worker, seven certified nursing assistants (CNAs), three bus drivers, and one social worker intern. Clients and CNAs were assigned to three different groups they called “homes.” Each “home” had two to three CNAs for 12 to 16 clients and was run as an independent unit.

*Center B* is run by a religious organization and aims to provide comprehensive quality of care with a person-centered approach. There were 57 clients enrolled with one director, one RN, two social workers, and seven CNAs. The clients were assigned to six groups based on their cognitive and physical abilities and gender. *Center B* was promoted for its long-standing history and family-like interactions between clients and staff.

*Center C* is operated by a senior citizen's welfare foundation valuing filial piety and promoting intergenerational love when caring for elders. There were 29 clients enrolled with one director, two RNs, one social worker, and four CNAs. The clients were assigned to three different groups based on their cognitive and physical abilities. In contrast to the other two centers where the CNAs normally stayed with the same group, the CNAs at *Center C* rotated among three groups every week. *Center C* was promoted for its convenient location and physical therapy equipment.

### 2.2. Participants

Participants were adults aged 18 years or older associated with study sites, including staff members, clients, interns, and volunteers. Staff participants were three center directors, five RNs, four social workers, 19 CNAs, two shuttle bus drivers, and one intern. Client participants were older adults who attended the center when the field observations were conducted. Interns and volunteers were included in the field notes because they assisted with leading activities and interacting with clients, which potentially could influence care delivery.

### 2.3. Data Collection

After obtaining permission of center directors to conduct the study, the first author scheduled three sequential weeks to conduct the investigation at each site. On the first day at each of the three centers, the first author provided an information session regarding the study for the staff and the clients. She was introduced by the director of each center not only as a researcher but also as a volunteer to help activities at the center. Each of the directors requested the first author to serve as a volunteer at their centers. Serving in this role facilitated the first author's immersion as a participant observer to build close relationships with staff that encouraged their openness during interviews. Participant observation was conducted at each center for 8 hours per day (from 9 am to 5 pm), 5 days a week for over three weeks at each center. Field notes were used to record the behaviors, conversations, and activities seen and heard in the field and also included observer's feelings and thoughts that arose during the fieldwork. If there were any indications such as signs of discomfort among the clients/staff or negative statements from a family member, the first author left the room and no notes were taken.

All staff members were personally invited to participate in interviews with the first author. Staff who orally agreed signed an informed consent form before the interview to indicate their understanding of the research process and to assure that their responses would be strictly confidential. Staff participants were given a questionnaire to complete before the interview. The questionnaire included measures of organizational culture and quality of care. Organizational culture was measured with the CVF for long-term care and assessed six dimensions: organizational characteristic, administration, management style, organizational glue, strategic emphasis, and criteria for success [17]. The CVF employs a Likert response scale where respondents are asked to rank from 1 (agree the least) to 4 (agree the most) across four response options, with 4 representing the characterization that best reflects how things work in their center [14]. Each culture type would have scores ranging from 6 to 24. The CVF assessment was translated into Mandarin Chinese by working with ADS staff and experts to assure the items were appropriately translated.

Assessment of quality of care, following the studies of Van Beek and Gerritsen [17] and Chung [27], was measured by asking staff members to choose the most important out of twelve tasks at center and to check the best description out of seven statements that constitute “good care.” The twelve tasks in the questionnaire were creating safe and clean surrounding, observing changes in the clients' condition, following restricted procedures (e.g., clients are not able to smoke at center or clients have to ask permission for medication), supporting clients emotionally, educating family members on disease process and behavior management, supporting family members emotionally, keeping care plans, creating a nice and friendly atmosphere, engaging in activities with clients, getting family members involved in daily life, stimulating social engagement of clients, and delivering personal individual care. The seven statements of good care included in the questionnaire were keep clients' body clean, keep clients happy, put yourself in the shoes of clients, keep communicating with your clients respectfully, be patient with clients, build up close relationships with clients, and keep clients safe.

Of the 31 staff participants who signed the informed consent and completed the questionnaire, four staff members decided to withdraw from the interviews due to personal reasons. The remaining 27 staff participants chose to complete the interviews at their centers because it was convenient. Open-ended interviews with staff were semistructured and started with a board question, such as, “Can you tell me about your previous experiences working with older adults?” Questions gradually became more focused and addressed the culture and care issues, such as, “Can you tell me about the culture at this center and how care is delivered to the clients? What do you think constitutes good care for people with dementia and why do you believe so?” Each interview was approximately one hour in duration and was conducted in a private room at center. The interviews were audio-recorded, transcribed, and translated verbatim by the first author.

### 2.4. Data Analysis

Quantitative data from 31 staff questionnaires were first recorded into an Excel spreadsheet to create a culture profile for each center in order to examine its dominant cultural type, the most important caregiving task, and what constitutes good quality of care. We then entered the data from the CVF assessment into IBM SPSS Statistics 25 to run a further analysis with ANOVA to compare the differences within and among centers.

Qualitative analysis was started when all 45 field notes were typed up, and all 27 staff interviews were transcribed and translated. The second author helped to check the English translation of the transcripts to ensure the validity. Both authors then triangulated the observational field notes with interview transcriptions and read them carefully to obtain a general sense of the information and to reflect on its overall meaning [28]. Authors coded all transcriptions independently center by center with content analysis by searching for meaning units (i.e., words, sentences, or paragraphs) related to center's culture, care delivery, and/or staff-staff/staff-client interactions. Finally, we met together to examine each code and discuss which ones to keep and which ones to delete/modify. After reaching consensus on all of the codes, we reorganized the codes by combining similar codes under broader categories to compare among three centers [26].

## 3. Results

The results of this study are organized into two sections. Descriptions of the cultural values conveyed on the CVF and the results of ANOVA are first introduced. The ethnographic data analysis follows with the quantitative findings triangulated from the field observation, staff interviews, and the first author's reflections as an observer.

### 3.1. Quantitative Data Analysis

#### 3.1.1. Staff Background and Caregiving Priorities

A large number of the staff participants (88%) obtained either a high school degree or a bachelor's degree, and the majority (65%) had prior experience with elderly care either in nursing homes (38%) or in community-based services (27%). All of the full-time staff worked 40 to 45 hours a week with an average 3.9 years of duration of working at the center.

Staff feedback on the most important caregiving tasks at their center and what constitutes good care are presented in Table 2. “Creating safe and clean surrounding” was reported by the largest number of staff (37%), followed by “delivering personal individual care” (26%). There was slightly more agreement among staff feedback on the best description what of constitutes “good care,” with 41% reporting that “good care was putting yourself in the shoes of the client,” followed by keeping clients happy (26%).

#### 3.1.2. Results of the CVF Assessment and ANOVA

The results of the CVF assessment were consistent with previous studies that the group culture had the strongest (on average mean = 20.18; SD = 2.64) and the market culture had the lowest (mean = 13.09; SD = 3.81). This difference in cultural values was significant (*p* < 0.01).

A one-way between-subjects ANOVA was conducted to determine differences between the three centers based on staff perception of their centers' culture values. There was a significant difference with *Center A*'s staff perceiving more of a market and hierarchy culture value at their center compared with the other two centers with *p* < 0.05 (*F* (2, 31) = 4.90, *p*=0.014, for market culture; *F* (2, 31) = 4.43, *p*=0.020, for hierarchy culture).

Although the group culture value was found to be the dominant cultural value across all three centers, there were some discrepancies between the administrative staff (including director, nurses, and social workers) and the CNAs. For example, more ADS administrative staff (73%) scored higher on group culture than their direct care workers (68%). A two-way ANOVA was conducted that examined the effect of center and job title on staff perception on organizational culture; however, due to the small sample size, there was no statistically significant interaction between the effects of center and job title on culture perception, *F* (2, 7) = 1.803, *p*=0.145.

Looking at differences within *Center A* and *Center B*, results revealed that all of their administrative staff (100%) scored highest on group culture, whereas only one-third of their CNAs (33%) had the same perception. In *Center C*, 75% CNAs scored highest on group culture, whereas only one administrative staff member scored highest on group culture.

To analyze the CVF flexibility scores for each center, we added the overall group and developmental cultural value scores. The higher the flexibility score, the more likely the center will have the capacity to create and sustain improvement [14]. Results of the three centers flexibility scores: *Center A* had the highest score of 37.36, *Center B* got the middle scores as 35.25, and *Center C* obtained the lowest as 33.25.

The results from the CVF assessment, however, may not show the whole story because staff may modify their answers to be more politically correct or correspond to Taiwanese culture [29]. Staff participants, for example, may modify their answers to align with how they think their center administrators would like them to respond rather than how they really feel about their workplace. In light of the possibility of subject bias, we also collected qualitative data with observation and interviews to provide the most accurate picture of the existing cultures of the three centers.

### 3.2. Qualitative Data Analysis

Although the CVF results showed that the three ADS centers had the group culture value as their dominant culture value, the observational field notes and staff interview transcripts demonstrated different care practices among the three centers. That is, each center may possess some elements of group culture (i.e., flexible management, teamwork, cohesion, employee concerns and ideas, and open discussion) but also have some nongroup cultural elements (i.e., staff-centered caregiving, hierarchical relationships between staff and staff and staff and clients, and conflict care ideas) to create its unique subsystem.

#### 3.2.1. Flexibility with Staff-Centered in Center A

The most prominent group culture value performed at *Center A* was flexible management. The director emphasized during the interview that, “I am pretty flexible in terms of management. If elders or staff have any reasonable suggestions, I am happy to sit down with them to come up with a solution to make changes. … if staff want to make a change in care delivery, I will hold a meeting to discuss with all staff.” The director also empowered the staff members to make their own decisions and tried to not be involved or intervene too much. Many staff appreciated this flexibility by saying: “I am happy working here. The director gives us lots of freedom and authority at center.”

The flexible management and empowering staff at *Center A*, however, led to a negative result. At *Center A*, the “we-ness” value from the group culture was replaced by “I-ness” or “staff-centeredness.” For example, the center director commented on CNAs that “some of the CNAs earned a second degree in aging care and believed that what they had learned from school was correct and did not accept social workers' or nurses' recommendations.” That is, some CNAs carried within themselves patterns of thinking that were learned from their degrees or previous experiences, and this influenced how they delivered the care and interacted with the clients at center.

Different views on caregiving led to different interactions with the clients. Using morning events from the field notes as an example: one morning during the exercise time, a male client requested to sit alone in the dining area, but the CNA leading the exercise refused his request and forced him to sit with the group in the living room. Another morning, the same male client requested to sit in the dining area during the morning exercise, and another CNA leading the exercise granted his request, so the male client sat alone in the dining area. There were many examples of CNAs performing caregiving differently. One CNA shared in the interview about her way of dealing with one client's problem behavior by describing that, “One of the clients has depression and sometimes disturbs the class …. To comfort her, I give her some vitamin C pills. But not all CNAs agree with me on feeding her vitamin C. Some CNAs just let her ask for medications for the whole class.” Another CNA disagreed with her coworkers' caregiving views by saying, “I did not agree with some coworkers' views. … they lead activities at center just like activities are led at child daycare. I think that elders are different from the children. Elders have lost some of their functions, so we do not push them too much because that will frustrate them more.”

The CNAs at *Center A* not only acknowledged their different caregiving ideas but also justified that theirs were the best by saying, “I am better than other CNAs because I know them [the clients] so well.” “I got an associate degree in elderly care. I learned a lot about how to provide care or lead activities for elders, so I apply what I learned at school to this job.” The “I-ness” also affected staff's views on what is the best characteristic for quality of good care. Among seven characteristics, 12 staff in *Center A* selected five different characteristics: keep clients happy, put yourself in the shoes of the client, keep clients safe, communicate with your clients respectfully, and build up close relationships with clients. Staff members' different views on good care influenced their interactions with clients and other coworkers. The head nurse shared in the interview that bullies existed among CNAs. Two senior CNAs had bullied one junior CNA due to different caregiving visions, so she had to intervene by reassigning those three CNAs in different units. This change created ripple effects on clients who were taken care of by those three CNAs and had to adjust to other CNAs taking care of them.

#### 3.2.2. Teamwork with Hierarchical Division in Center B

One of the group culture values emphasized in *Center B* was teamwork. The center director pointed out in the interview that, “Here we work as a team so if we find someone (staff member) who does not fit into our team, we may decide to let the person go.” One of the CNAs shared that “when I cannot calm my clients down, I will ask other CNAs to help. This is the teamwork that we support one another with.” However, the administration and other CNAs had different definitions on teamwork.

For the administration (including the director, the head nurse, and two social workers who were supervisors of the CNAs), the teamwork value was translated as they were the leaders of the team to plan and control the system at center, and the CNAs were under them and needed to implement their orders. The director stated that, “… the CNAs here are very good at fulfilling our [administration] demands …. when we shared with the CNAs changes about providing the night care, they worried at the beginning but still accepted them instead of quitting their jobs.” If the CNAs did not fully follow their orders, the administrative staff blamed CNAs' educational attainment: “most of the CNAs in this center just obtained an associate degree, so it is hard for us to communicate our views with them.” When the CNAs had inappropriate interactions with the clients, the administration focused on the CNAs' faults: “…they [CNAs] did not fully absorb or internalize the training information to practice at center and still used their old ways to interact with the clients.” “If clients are emotional, the CNAs need to be skillful to distract them. It is not right to yell at clients and be angry with them.”

CNAs expressed the gap existing between themselves and the administration, particularly on administration's lack of support and poor communication. They also acknowledged that they had to follow the directions from the administration as the top management and had no influence on any changes happening at center. Therefore, some of them had already given up on providing suggestions or asking for support from the administrative staff. In addition to the poor support from the administration, some CNAs addressed no hope to be promoted at center: “For the CNAs at center, there are very few ways to get a promotion. You have to get another degree in social work to move on to the administrative work.” Moreover, they also worried about losing their clients because “declining numbers of the clients in my group will affect my evaluation, which affects my salary.” In other words, the blame that the administration places on the CNAs for “losing” clients in their groups may result in administration giving those staff members a poor annual evaluation to reduce their salary raise. All of these pressures on the CNAs may lead them to feel overwhelmed and possibly cause negative interactions with clients at center.

The hierarchical division was not just between CNAs and the administration at the *Center B* but also existed between the CNAs and their clients due to fulfilling the idea of “group life” at center. The head nurse articulated that, “Our center emphasizes group life, so if one client does not want to stay in the group, the CNAs need to find a way to handle the client's behavior to keep them (within their group).” The social worker further addressed the rules for group life at center: “Elders here have to follow a group lifestyle. That is, we do not have a personalized schedule for each client at center so every client has to follow the same schedule.” In order to implement the group life at center, the CNAs were empowered to control clients' behavior. The director told the first author that “you might hear that CNAs describe themselves as teachers or asked the clients here to call them teachers. It is because we want to empower the CNAs. The teacher-student relationships can help the CNAs to manage the clients' behavior.” With the title of “teachers,” the CNAs perceived their roles as to “correct” clients' behavior or to help them “slow down” their degeneration. Some CNAs shared in the interviews that, “The elders here have some abnormal behavior due to their dementia. When I observe these abnormal behaviors, I will correct them.” “We teach many classes here at the center to help them [the clients] slow down dementia as well as keeping their abilities.”

When the CNAs focused too much on correcting elders' behavior, negative interactions occurred. The director explained that this happened because “CNAs are not good at putting themselves in clients' shoes. They need to empathize more with the clients.” This comment sounds ironic because the administration did not empathize with the CNAs. Responding to the survey question on what the best quality of care is, interestingly, half of the CNAs checked “put yourself in the shoes of the client.” It appears that CNAs at *Center B* had the knowledge of what is the best care but did not always perform it because they viewed themselves as teachers (rather than direct care workers) and older client are their students. As mentioned above, the teacher-student roles between the CNAs and the clients were promoted by the administration to empower CNAs to better manage the clients' behavior. However, the director commented in the interview that, “The teacher-student relationships may hinder the CNAs to treat their clients with dignity.” Other administrator staff also had similar comments on how the CNAs abused their power as the teachers and treated their clients as powerless students without giving them any positive feedback or supportive statements.

#### 3.2.3. Shared Value with Conflict in Center C

For the third center, we found that several care-related themes repeatedly emerged from the staff interviews. These included “treat the clients like family members,” “elders here like my parents,” and “my relationship with the elders here is personal.” In *Center C*, there was a shared purpose in providing good care and respecting the clients. To achieve this, there was consistent emphasis on the value of treating clients as your family members. One of the nurses stated in the interview that she learned this value from other staff: “I remembered that during my job interview here, the previous director told me that they want to create a home-like environment at center. After getting this job, I observed that the staff treat the clients like their family. Therefore, for me, I also see the clients here like my family.” Many CNAs also had learned this value of respecting clients by stating: “Here I learned that we cannot hurt the elders' dignity at center.” “We learned that we cannot force the elders to do activities.” “My principle of interacting with elders is respect. … We learned to treat elders the way we would like to be treated when we are old.”

Respecting elders in *Center C* was not just a shared value but also integrated into the care practice plan. The director shared that clients at center were just like her grandparents, so she wanted to help them maintain their abilities on performing activities of daily living (ADLs). To achieve this, she demanded the CNAs to do a perfect job with hands-on care and leading excellent activities. For nurses, they rotated the CNAs among four different client groups because they wanted the CNAs to “become the clients' family members.” One nurse further explained that, “If we required the CNAs to take care of the same group of the clients, they would not be able to know other clients at other groups. They also may develop a perspective of ‘your clients versus my clients' and not provide support to the clients that are outside their groups. Therefore, we teach the CNAs that we are all clients' family members. Rotation among client groups will help them to perform like family members.” For CNAs, they accepted and performed this value very well. From the field note record, it showed that they gave clients more autonomy to make decisions on participating activities and interacted with clients very respectfully without any scolding or yelling toward the clients.

All staff at *Center C* acknowledged the value of respecting clients; however, the director and the nurses expressed very different ideas on what is the best way to practice this value. The director emphasized, “a clear line for each staff's working responsibilities,” and hoped that the nurses would take more responsibilities on checking and examining the clients' physical conditions, so the CNAs would focus just on hands-on care works and leading activities. However, the nurses, the supervisors of the CNAs, focused on having CNAs pay close attention to each client's physical changes and report back to them in detail. Both nurses highlighted in the interview that the CNAs needed more training on chronic diseases and mental health in order to be more sensitive in detecting the clients' physical and emotional changes as well as being able to provide a precise report to them. The director and the nurses not only had different views on care practice but also distrusted each other. The director blamed the nurses for being unable to perform their jobs well by saying: “To be honest with you, I do not see that we have two nurses performing their professions well. The nurses always just ask the CNAs to report clients' conditions but do not go to check with the clients on themselves.” One nurse highlighted the difference: “I feel so frustrated communicating with other administrators because they are social workers and do not understand our concerns. … they do not trust you …” Another nurse referred to the nursing versus social work background at center: “For most of the time, the higher administrators will go along with our ideas, but sometimes they do not agree with us and force us to follow their ideas. Then it becomes hard for us to communicate with them due to coming from different disciplines.”

Although there were conflicting views within the administration, the CNAs were not affected and were still able to provide quality of care at center. They might get confused about the main caregiving goal at center but tried to fulfill both requirements on leading activities and observing clients' physical changes. The CNAs did recognize that working at the center was “a tough job” or “a labor and mentally demanding job.” However, they also recognized that they received “the full support from the administration, from both the nurses and social worker,” and from the training classes, so they “were equipped to work with different people” and “felt happy to work at center.” The CNAs at *Center C* were always busy either leading activities or checking the clients' physical conditions and filling out the record forms but still were very patient with the clients. The CNAs appeared to treat the clients nicer than their family members. The first author witnessed several times that the clients' family caregivers verbally bullied the clients, and the CNAs intervened to stop this mistreatment.

## 4. Discussion

Organizational culture has been recognized as affecting the quality of care and behavior and attitudes of staff across health and social care settings [16, 17]. Supporting previous research done with the CVF assessment at nursing homes, quantitative analyses revealed that the group culture was the predominant culture at each of the three ADS centers. The evidence from the qualitative data, however, revealed dynamic interaction at each center, which created significant variation and a particular care culture for each center.

This study also identified three different subsystems under the group culture values derived from the ethnographic data. *Center A* demonstrated the group culture value of flexible management, and this value shaped the care practice and enabled staff to act autonomously. However, staff at *Center A* did not possess a shared purpose and performed self-determined caregiving very differently, which led to negative results. *Center B* demonstrated the group culture value of teamwork with CNAs supporting one another. The administrative staff, however, recognized teamwork in “vertical,” hierarchical terms rather than as a “flat,” collaborative structure [30]. Therefore, achieving the goal of good teamwork was hindered by inadequate communication between administration and CNAs, and this vertical team value was then reflected with the negative interactions between the CNAs and the clients at center. *Center C* demonstrated the group culture of a shared value on respecting clients, which was performed on the day-to-day basis. Although there was a conflict view within management, it did not affect the good care provided in *Center C*. Overall, each of three centers demonstrated three different subsystems and mirrored previous studies concluding that organizational culture is a very complex dynamic and a locally developed phenomenon [15–17].

Survey results revealed that staff at each of the three centers perceived more group culture values than the other three cultural values (i.e., developmental culture, hierarchical culture, and market culture). This finding may reflect a Taiwanese cultural effect. The traditional Taiwanese culture emphasizes collectivism and solidarity within the group that manifests in a commitment to in-group members with a strong relationship and support of each other [31, 32]. In collective societies, socialization places emphasis on compliance, obedience, and responsibility [33]. ADS staff surveyed in this study grew up with this collective culture and, thus, seemed comfortable to adapt group culture values at their center. However, some collectivist values may conflict with the group culture values. Take power distance for example. Collectivists value power distance and believe that lower-level members of institutions have to defer to members who are more powerful, which could lead to supervisor-subordinate relationships [34]. Therefore, people living and working within a collective culture background may easily expect and accept that power is not shared equally between subordinate and supervisor [34]. The first author did observe unequal power sharing between the administration (supervisor) and the CNAs (subordinate) in all three centers but more so at *Center B*. Furthermore, the first author noticed the hierarchical relationships between CNAs (supervisor) and the clients (subordinate). Although the CNAs at *Center B* complained about the power distance between them and administrators at center, they also displayed such behaviors while delivering care and interacting with the clients at center. In line with Wicke et al.'s [30] study, the power distance led to hierarchical relationships that led to a significant barrier to effective teamwork and quality of care at *Center B*.

Although previous research conducted in the nursing homes concludes that the group culture was positively correlated to the good quality of care [14, 17, 24], this study showed a combination of positive and negative consequences of practicing group culture values. The group culture value stresses the empowerment of the employees and emphasizes employee development as driving the care delivery in the health care settings. Research in the long-term care facilities in Taiwan has shown that organizational empowerment is significantly associated with total job satisfaction among CNAs [35]. For this study, staff empowerment led to high staff retention. Specifically, the majority of staff members at three centers had a long working history with 14 years as the longest duration of employment at a center. Staff expressed their enjoyment working at center due to the authority and flexibility they obtained. However, empowering staff may result with some problems. For example, at *Center A,* some CNAs delivered the care based on their individual judgment, so the care delivery to the same client varied based on which CNA took care of him/her. For people with dementia who need a routine, inconsistent care plans may confuse them and affect their quality of life at the center. Moreover, CNAs at *Center B* were empowered to control clients' behavior; however, the high demand on control at center failed to respect each client's differences and led to negative interactions when the clients were perceived as “out of staff's control.” It is important to find the balance between an empowered workforce and respectful treatment toward the clients. Empowering not only staff but also clients should be considered as a strategy of providing quality of care at ADS [36].

### 4.1. Strengths, Limitations, and Directions for Future Research

In contrast to previous research on organizational culture in healthcare settings where data were collected with either a quantitative or a qualitative method, this study is one of the few to include a mixed method to provide a more comprehensive perspective at ADS centers. Organizational culture may be difficult to identify and assess by quantitative research methods alone because the results may only reflect the “public” voice of participants and not their “private” voice [37, 38]. However, the use of individual interviews and participant observation in this study provided insight on underlying shared values, beliefs, assumptions, and norms at ADS centers, which are difficult to identify and assess by quantitative research methods alone [37]. This study used data from both questionnaire responses from staff and extended observation and interviews to explore connections and disconnections between the culture participants reported in the questionnaire and the actual culture they participated in from the observations. Moreover, the breadth of participants, including both staff and clients, increases the reliability of results. A limitation is that data were collected at three sites in one large urban city, so the results of this study may not be necessarily generalizable to ADS centers in small cities and rural areas of Taiwan. Future research may consider collecting data from a larger number of ADS in Taiwan in order to increase statistical power when examining the impact of organizational cultural on care performance.

In addition, using only staff self-reported survey data on organizational culture has its bias. Staff participants in this study might have answered the questionnaire in a way to make a positive impression on others as well as themselves [39]. To fully understand the nuances of the organizational culture, future research may consider inviting third parties visiting ADS clients, such as family members or close friends, to take the CVF survey and answer questions on caregiving priorities. This study was also limited to the ADS staff's interviews, and future studies may benefit by including clients' voices, which have been proved crucial contributors to the content, process, and outcomes of care as well as a good way to empower the clients for social engagement [40, 41].

Finally, based on her familiarity with the three ADS centers in her hometown, the first author did not anticipate observing mistreatment of the clients and was not prepared to intervene. Furthermore, members from her sponsoring university's Human Subjects Protection Committee reported no concerns regarding the nature of study or the potential of witnessing mistreatment at the ADS centers. The first author has researched Taiwanese elder abuse laws and developed maltreatment intervention procedures that will be included in future research proposals submitted to the university's Human Subjects Protection Committee.

Despite these limitations, this study is the first to examine organizational culture in ADS in Asia. Optimally, the rich descriptive data drawn from the in-depth participant observation and the interviews lay the foundation for future researchers, policy makers, and practitioners to build upon.

## 5. Conclusions

Taiwan is becoming an aged society; however, the Taiwan government seems lacking in endeavors on aging research, particularly on the quality of care at long-term care facilities [1]. In response to the challenge of the rapidly growing older population, more research on senior care services is required to guide the policies and practices to meet the needs of the aging population in Taiwan. Previous studies in healthcare setting have reported associations between organizational culture and care delivery, quality of care, and residents'/patients' experiences [12, 16–21]. This study is one of the few done at ADS centers in Taiwan to demonstrate the linkages between organizational culture and care performance. As ADS are highly encouraged and sponsored by the Taiwan government, future research is necessary to examine the relationship between care quality and organizational culture at more ADS centers in Taiwan.

Previous studies have shown that nurses/nursing assistants in long-term care facilities who view their work environments as empowering are more likely to provide high-quality care and experience satisfaction with their jobs, which led to positive resident outcomes [35, 42, 43]. Although the results of our study indicated that CNA empowerment at ADS centers not only contributed to low turnover and high retention but also contributed to negative workplace and client interactions. For example, although administrators at *Center A* and *Center B* did not intend to create negative care environments through empowering CNAs, these CNAs (empowered with a staff-centered care philosophy or with a teacher status) believed that security was a top priority and created a hierarchical atmosphere, which decreased the autonomy and quality of life of their clients [44]. It is critical that future researchers and practitioners identify ways to balance empowering staff, while ensuring clients' security, autonomy, and quality of life at ADS in Taiwan and other Asian countries possessing similar cultural traditions and population trends.

## Figures and Tables

**Table 1:** Three centers' and its clients' information.

	*Center A*	*Center B*	*Center C*
Center information			
Ownership	Private, nonprofit	Private, nonprofit	Private, nonprofit
Funding (%)			
Government support	62%	89%	86%
Private pay	38%	19%	14%
Cost of participation			
Per day	$33	$33	$27
Per month	$500	$500	$500
Client information			
Total enrolled	50	57	29
With Alzheimer's disease only	46%	74%	79%
With physical disability only	24%	35%	21%
With Alzheimer's and physical disability	30%	46%	0%
With depression	4%	9%	0%

**Table 2:** Scores of CVF and perceptions on task priorities and quality of care among staff participants at three ADS centers.

	Group	Development	Market	Hierarchy	Most important task	Quality of good care
*Center A*						
Center director	22	18	13	17	Creating a nice and friendly atmosphere	Keep clients happy
RN 1	23	19	19	22	Creating safe and clean surrounding	Put yourself in the shoes of the client
RN 2	23	20	19	18	Creating safe and clean surrounding	Keep clients safe
Social worker	20	12	14	14	Creating safe and clean surrounding	Keep clients safe
CNA 1	23	18	18	18	Creating safe and clean surrounding	Put yourself in the shoes of the client
CNA 2	18	16	17	18	Creating safe and clean surrounding	Put yourself in the shoes of the client
CNA 3	21	21	13	14	Creating a nice and friendly atmosphere	Put yourself in the shoes of the client
CNA 4	23	20	19	18	Creating safe and clean surrounding	Keep clients safe
CNA 5	19	17	19	17	Delivering personal individual care	Communicate with your clients respectfully
CNA 6	17	12	16	17	Delivering personal individual care	Keep clients happy
CNA 7	22	12	11	19	Stimulating social engagement of clients	Build up close relationships with clients
CNA 8	15	14	13	18	Creating safe and clean surrounding	Keep clients happy
Driver 1	22	12	11	18	Educating family members on disease process and behavior management	Keep clients happy
Driver 2	22	19	19	22	Observing changes in clients' conditions	Put yourself in the shoes of the client
Intern	20	7	12	21	Observing changes in clients' conditions	Keep clients happy
Mean	20.29	15.43	15.21	18		

*Center B*						
Center director	24	13	10	13	Delivering personal individual care	Keep clients happy
Nurse	21	14	10	13	Delivering personal individual care	Keep clients happy
Social worker	23	14	8	15	Creating a nice and friendly atmosphere	Put yourself in the shoes of the client
CNA 2	21	21	16	19	Observing changes in the clients' condition	Build up close relationships with clients
CNA 3	15	17	8	16	Delivering personal individual care	Put yourself in the shoes of the client
CNA 4	16	21	9	14	Observing changes in clients' conditions	Put yourself in the shoes of the clients
CNA 5	20	14	9	14	Creating a nice and friendly atmosphere	Keep clients happy
CNA 6	18	10	15	17	Supporting client emotionally	Put yourself in the shoes of the clients
Mean	20.25	15.17	11.83	16.08		

*Center C*						
Center director	20	16	13	23	Delivering personal individual care	Put yourself in the shoes of the client
Nurse 1	15	11	18	10	Delivering personal individual care	Put yourself in the shoes of the client
Nurse 2	17	13	12	18	Creating safe and clean surrounding	Build up close relationships with clients
Social worker	21	15	9	14	Creating safe and clean surrounding	Keep clients clean and happy
CNA 1	24	15	6	11	Observing changes in the clients' condition	be patient with clients
CNA 2	22	15	8	15	Observing changes in the clients' condition	Put yourself in the shoes of the client
CNA 3	17	13	12	18	Creating safe and clean surrounding	Build up close relationships with clients
CNA 4	23	9	9	12	Creating safe and clean surrounding	Put yourself in the shoes of the clients
Mean	19.88	13.63	11	15.5		

## Data Availability

The quantitative data used to support the findings of this study are available from the first author upon request. The institutional review board restricts the qualitative data used to support the findings of this study in order to protect participants' privacy. Data are only available for researchers who meet the criteria for access to confidential data.

## References

[1] Lin Y.-Y., Huang C.-S. (2016). Aging in Taiwan: building a society for active aging and aging in place. *The Gerontologist*.

[2] (2016). The ten year long-term care project 2.0. https://1966.gov.tw/LTC/cp-3636-38462-201.html.

[3] Chen Y.-J. (2008). Strength perspective: an analysis of ageing in place care model in Taiwan based on traditional filial piety. *Ageing International*.

[4] Lin L.-C., Wang T.-G., Chen M.-Y., Wu S.-C., Portwood M. J. (2005). Depressive symptoms in long-term care residents in Taiwan. *Journal of Advanced Nursing*.

[5] Chiu H.-C. (2002). Organization and delivery of long-term care in Taiwan. *Journal of Aging & Social Policy*.

[6] (2014). Taiwan caregiving planning. https://www.ey.gov.tw/News_Content.aspx?n=F8BAEBE9491FC830&s=8CE2B4DD55E167AD%E3%80%82.

[7] Lu P.-C. (1997). The effect of day care services in Taiwan: exploring users’ view (In Chinese). *The NCCU Journal of Sociology*.

[8] Chang D.-S., Yang S.-L. Combining Kano model and service blueprint for adult day care service—A case study in Taiwan.

[9] Lin L.-J., Li K.-Y., Tabourne C. E. S. (2011). Impact of the life review program on elders with dementia. *Journal of Nursing Research*.

[10] Spilsbury K., Hewitt C., Stirk L., Bowman C. (2011). The relationship between nurse staffing and quality of care in nursing homes: a systematic review. *International Journal of Nursing Studies*.

[11] Brittis S. (2011). Humane care: seeing the person behind the patient-A case study. *Care Management Journals*.

[12] Nakrem S. (2015). Understanding organizational and cultural premises for quality of care in nursing homes: an ethnographic study. *BMC Health Services Research*.

[13] Custers A. F. J., Kuin Y., Riksen-Walraven M., Westerhof G. J. (2011). Need support and wellbeing during morning care activities: an observational study on resident-staff interaction in nursing homes. *Ageing and Society*.

[14] Scott-Cawiezell J., Jones K., Moore L., Vojir C. (2005). Nursing home culture. *Journal of Nursing Care Quality*.

[15] Scott T., Mannion R., Marshall M., Davies H. (2003). Does organisational culture influence health care performance? A review of the evidence. *Journal of Health Services Research & Policy*.

[16] Killett A., Burns D., Kelly F. (2016). Digging deep: how organisational culture affects care home residents’ experiences. *Ageing and Society*.

[17] Van Beek A. P. A., Gerritsen D. L. (2010). The relationship between organizational culture of nursing staff and quality of care for residents with dementia: questionnaire surveys and systematic observations in nursing homes. *International Journal of Nursing Studies*.

[18] Alharbi T. S. J., Ekman I., Olsson L.-E., Dudas K., Carlström E. (2012). Organizational culture and the implementation of person centered care: results from a change process in Swedish hospital care. *Health Policy*.

[19] Carney M. (2011). Influence of organizational culture on quality healthcare delivery. *International Journal of Health Care Quality Assurance*.

[20] Etherton-Beer C., Venturato L., Horner B. (2013). Organizational culture in residential aged care facilities: a cross-sectional observational study. *PLoS One*.

[21] Lyons S. S. (2009). How do people make continence care happen? An analysis of organizational culture in two nursing homes. *The Gerontologist*.

[22] Kitwood T. (1997). *Dementia Reconsidered: The Person Comes First*.

[23] MacLachlan M. (2006). *Culture and Health: A Critical Perspective towards Global Health*.

[24] Banaszak-Holl J., Castle N. G., Lin M., Spreitzer G. (2013). An assessment of cultural values and resident-centered culture change in U.S. nursing facilities. *Health Care Management Review*.

[25] Quinn R. E., Rohrbaugh J. (1981). A spatial model of effectiveness criteria: toward a competing values approach to organizational analysis. *Management Science*.

[26] Chuang Y.-H., Abbey J. (2009). The culture of a Taiwanese nursing home. *Journal of Clinical Nursing*.

[27] Chung G. (2012). Understanding nursing home worker conceptualizations about good care. *The Gerontologist*.

[28] Creswell J. W. (2009). *Research Design: Qualitative, Quantitative, and Mixed Methods Approaches*.

[29] Cherry K. E., Allen P. A., Denver J. Y., Holland K. R. (2015). Contributions of social desirability to self-reported agesim. *Jounral of Applied Gerontology*.

[30] Wicke D., Coppin R., Payne S. (2004). Teamworking in nursing homes. *Journal of Advanced Nursing*.

[31] Hofstede G., Hofstede G. J. (2005). *Cultures and Organizations: Software of the Mind*.

[32] Tang H.-W. V., Yin M.-S., Nelson D. B. (2010). The relationship between emotional intelligence and leadership practices: a cross-cultural study of academic leaders in Taiwan and the USA. *Journal of Managerial Psychology*.

[33] Ali A. J., Lee M., Hsieh Y. C., Krishnan K. (2005). Individualism and collectivism in Taiwan. *Cross Cultural Management: An International Journal*.

[34] Patterson P. G., Buranapin S., Kantabutra S. (2015). Frontline employees’ cognitive appraisals and well-being in the face of customer aggression in an Eastern, collectivist culture. *Journal of Services Marketing*.

[35] Kuo H.-T., Yin T. J.-C., Li I.-C. (2007). Relationship between organizational empowerment and job satisfaction perceived by nursing assistants at long-term care facilities. *Journal of Clinical Nursing*.

[36] Tu Y.-C., Wang R.-H., Yeh S.-H. (2005). Relationship between perceived empowerment care and quality of life among elderly residents within nursing homes in Taiwan: a questionnaire survey. *International Journal of Nursing Studies*.

[37] Hesselink G., Vernooij-Dassen M., Pijnenborg L. (2013). Organizational culture. *Medical Care*.

[38] Kirkley C., Bamford C., Poole M., Arksey H., Hughes J., Bond J. (2011). The impact of organisational culture on the delivery of person-centred care in services providing respite care and short breaks for people with dementia. *Health & Social Care in the Community*.

[39] Shinan-Altman S., Cohen M. (2009). Nursing aides’ attitudes to elder abuse in nursing homes: the effect of work stressors and burnout. *The Gerontologist*.

[40] Dabelko-Schoeny H., King S. (2010). In their own words: participants’ perceptions of the impact of adult day services. *Journal of Gerontological Social Work*.

[41] Kehyayan V., Hirdes J. P., Tyas S. L., Stolee P. (2015). Residents’ self-reported quality of life in long-term care facilities in Canada. *Canadian Journal on Aging/La Revue canadienne du vieillissement*.

[42] Barry T. T., Brannon D., Mor V. (2005). Nurse aide empowerment strategies and staff stability: effects on nursing home resident outcomes. *The Gerontologist*.

[43] Ning S., Zhong H., Libo W., Qiujie L. (2009). The impact of nurse empowerment on job satisfaction. *Journal of Advanced Nursing*.

[44] Salari S. M. (2006). Infantilization as elder mistreatment: evidence from five adult day centers. *Journal of Elder Abuse & Neglect*.

